# Intravaginal practices and microbicide acceptability in Papua New Guinea: implications for HIV prevention in a moderate-prevalence setting

**DOI:** 10.1186/1756-0500-5-613

**Published:** 2012-11-01

**Authors:** Andrew Vallely, Lisa Fitzgerald, Voletta Fiya, Herick Aeno, Angela Kelly, Joyce Sauk, Martha Kupul, James Neo, John Millan, Peter Siba, John M Kaldor

**Affiliations:** 1 The Kirby Institute, The University of New South Wales, Sydney, NSW, 2052, Australia; 2 Papua New Guinea Institute of Medical Research, Goroka, Papua New Guinea; 3 School of Population Health, University of Queensland, Brisbane, Australia; 4 International HIV Research Group, School of Public Health and Community Medicine, University of New South Wales, Sydney, Australia; 5 HOPE Worldwide, Port Moresby, Papua New Guinea; 6 Sexual Health and Disease Control Branch, National Department of Health, Port Moresby, Papua New Guinea

**Keywords:** Vaginal microbicide, Acceptability, HIV prevention, Papua New Guinea

## Abstract

**Background:**

The acceptability of female-controlled biomedical prevention technologies has not been established in Papua New Guinea, the only country in the Pacific region experiencing a generalised, moderate-prevalence HIV epidemic. Socio-cultural factors likely to impact on future product uptake and effectiveness, such as women’s ability to negotiate safer sexual choices, and intravaginal hygiene and menstrual practices (IVP), remain unclear in this setting.

**Methods:**

A mixed-method qualitative study was conducted among women and men attending a sexual health clinic in Port Moresby. During in-depth interviews, participants used copies of a hand-drawn template to indicate how they wash/clean the vulva and/or vagina. Interviewers pre-filled commercially available vaginal applicators with 2-3mL KY Jelly® to create a surrogate vaginal microbicide product, which was demonstrated to study participants.

**Results:**

A total of 28 IDIs were conducted (women=16; men=12). A diverse range of IVP were reported. The majority of women described washing the vulva only with soap and water as part of their daily routine; in preparation for sex; and following sexual intercourse. Several women described cleaning inside the vagina using fingers and soap at these same times. Others reported cleaning inside the vagina using a hose connected to a tap; using vaginal inserts, such as crushed garlic; customary menstrual ‘steaming’ practices; and the use of material fragments, cloth and newspaper to absorb menstrual blood. Unprotected sex during menstruation was common. The majority of both women and men said that they would use a vaginal microbicide gel for HIV/STI protection, should a safe and effective product become available. Microbicide use was considered most appropriate in ‘high-risk’ situations, such as sex with non-regular, transactional or commercial partners. Most women felt confident that they would be able to negotiate vaginal microbicide use with male sexual partners but if necessary would be prepared to use product covertly.

**Conclusions:**

Notional acceptability of a vaginal microbicide gel for HIV/STI prevention was high among both women and men. IVP were diverse in nature, socio-cultural dimensions and motivators. These factors are likely to impact on the future acceptability and uptake of vaginal microbicides and other biomedical HIV prevention technologies in this setting.

## Background

Women bear a disproportionate burden of HIV, constituting over 50% of all those living with HIV worldwide and nearly 60% in sub-Saharan Africa [1]. In many countries, gender inequity means that women are unable to negotiate condom use, especially within marriage. Safe, effective, affordable HIV prevention methods that are initiated and controlled by women have therefore been seen as a high priority for HIV prevention. Vaginal microbicides are gels, cream or other topical preparations applied by a woman to reduce her risk of acquiring HIV sexually [2]. Some preparations have been designed for application immediately before and/or after each vaginal sex act (e.g. gels, dispersible tablets, films), whilst others such as intravaginal rings are intended to be placed in the vagina and to release microbicide over several weeks, providing protection independent of the timing of sexual activity. The past two decades have seen a number of trials of candidate microbicides, but until recently, none had been proven to be safe and effective in clinical trials [3-5]. A major breakthrough occurred in 2010 when the CAPRISA 004 trial in South Africa found that vaginally-administered tenofovir gel reduced HIV acquisition in women by 39% overall (and by 54% in those with high adherence), and was safe, acceptable and well tolerated [6]. Furthermore, the gel provided a similar level of protection to women against acquisition of *Herpes simplex* type-2 (HSV-2). Should these results be confirmed in a second trial [7], a new era in HIV prevention can truly begin.

The availability of a new prevention technology however, raises new questions for public health policy makers. In order for tenofovir gel and other tenofovir-based microbicides currently in development (such as vaginal films and dispersible pessaries [8-10]) to have the greatest impact, research is required to understand the diverse socio-cultural, behavioural and structural contexts into which such products could be introduced [11]. Information is also required on their acceptability to women and their sexual partners, and the impact that vaginal microbicide use may have on other HIV prevention strategies, particularly condom acceptability and use [11-14]. For example, research in Africa showed that although vaginal microbicides were conceived as female controlled methods that could be used covertly and not require male consent, in practice most women inform their partners, showing that acceptability and use need to be seen in the context of couple relationships [13]. The same research further showed how cultural preference for specific sexual practices interacted with microbicide acceptability [15]. A study among female sex workers (FSWs) in Uganda [16] found that foaming microbicide tablets and sponges were the most popular delivery mechanisms, and gel and film the least. Participants found the gel too messy and worried that the film would not dissolve. They liked the sponges because they could insert them in the morning and would be prepared for unexpected sex. Alternatively, in the MDP301 trial [4], married women in the same area of Uganda were very positive about study gel because it enhanced sexual enjoyment due to increased lubrication. It is possible that these differences were related to the different kinds of sexual relationships that the women in the two studies had and/or to the different physical properties of the two gels. This suggests that different products will be preferred in different contexts, and that acceptability research is necessary in different potential target groups.

Intravaginal hygiene and menstrual practices (IVP) have been recognised as possible risk factors for HIV and STI acquisition in women [17-23] and important influences on the acceptability and efficacy of vaginal microbicides in HIV prevention trials [24-28]. Research from a variety of developed and developing countries indicates that IVP are common among women at different levels of HIV/STI acquisition risk [15,17,24,27-35]. For example, in a study among women working in food and recreational facilities in Mwanza, Tanzania, Allen et al. (2010) found that intravaginal cleansing using fingers, water and soap was considered necessary to remove vaginal secretions, menstrual blood and post-coital discharge, and was carried out within 2 hours of 45% of reported sex acts [24]. A household survey among 3610 women in Mozambique, South Africa, Indonesia and Thailand found that the prevalence, type, frequency, and motivations for IVP varied significantly by setting, with intravaginal cleansing and insertion of traditional products most common in African study sites and oral ingestion of products considered to have vaginal effects most common in Asia [29]. IVP have the potential to negate the protective efficacy of vaginal microbicides for HIV prevention either by creating an adverse intravaginal environment (e.g. by altering intra-luminal pH) or by mechanical means (e.g. microbicide gel is washed out immediately after sex) [24]. They are also likely to impact on the acceptability and preference of different microbicide products in different settings (e.g. gels may be preferred in settings where vaginal ‘wetness’ is desirable and where women enhance lubrication through IVP; intravaginal rings or film formulations may be preferred in settings where women are concerned about partner perceptions of ‘excessive’ vaginal lubrication and where they use IVP to induce a ‘drier’ vagina prior to sex) [27,35,36].

Although overall HIV prevalence is below 1% in virtually all Asia-Pacific countries, the region is second only to sub-Saharan Africa in terms of the number of people living with HIV [37]. The HIV epidemic in Asia has been largely concentrated in people who inject drugs, sex workers and their clients and men who have sex with men, but increases in heterosexually-acquired HIV infection have been reported from a number of countries, including Bangladesh [38,39], China [40,41], Indonesia [42-44] and Pakistan [45,46]. Of particular note has been the increase in the proportion of women living with HIV in the region from 19% in 2000 to 35% in 2008, with striking increases observed in China [40] and India [47]. There are emerging, generalized heterosexual HIV epidemics in PNG and in neighbouring Tanah Papua Province in Indonesia, with estimates of adult HIV prevalence in the range 1-2% in these settings [48]. Other countries in the Pacific have to date experienced modest HIV epidemics but very high prevalences of sexually transmitted infections (STIs) have been reported both among FSWs and in community-based surveys [48-53]. The HIV epidemic in PNG is primarily linked to heterosexual transmission and shows significant heterogeneity, with over half the reported HIV diagnoses coming from three of its 22 provinces [54], placing women in some locations and socio-cultural contexts at considerably higher risk of HIV infection than others [48,54-58]. Some models have forecast that under certain scenarios, adult HIV prevalence in PNG could reach 10% over the next 15 years [59,60]. In Indonesia, Tanah Papua Province is experiencing a generalized HIV epidemic that has many parallels with the epidemic in neighbouring PNG. HIV prevalence among men and women aged 15–49 years in Tanah Papua was recently estimated at 2.4%, the highest in Indonesia and approximately 15 times the national average [61]. Among ethnic Papuans, HIV prevalence was almost twice that of other residents (2.8%, 1.5% respectively).

In these settings, new, female-initiated HIV prevention agents are potentially very appealing to both women at risk and to public authorities responsible for prevention. Apart from some limited trials of earlier candidate microbicides in two sites in Thailand [62-66] and India [66-72], virtually all of the recent trials of microbicides have been conducted in African countries. Asia-Pacific countries have been considered to have HIV incidence that is too low to justify efficacy trials, but high enough in some areas for microbicides to play an important role in prevention once they are proven safe and efficacious.

In this paper we report findings from the first study conducted in a moderate-prevalence setting in the Pacific region to investigate intravaginal practices and vaginal microbicide acceptability, and discuss the implications of our findings on future HIV prevention policy and research priorities.

## Methods

This study was conducted over a nine-month period in 2010–11. Fieldwork and data collection were carried out from April – June 2010, followed by the interview data transcription, translation, coding and qualitative data analysis.

**Figure 1:**
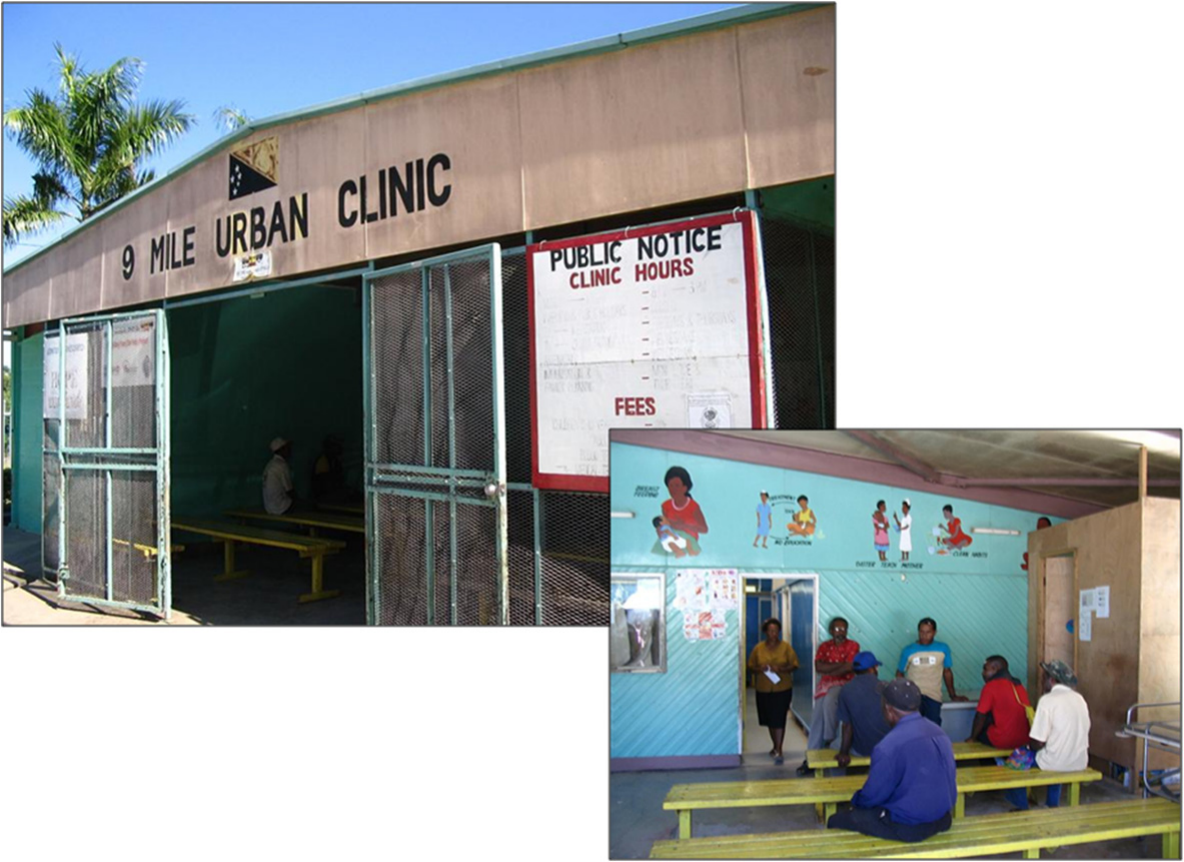
Nine-Mile Sexual Health Clinic, Port Moresby, PNG.

The study was conducted at Nine-Mile Sexual Health Clinic in Port Moresby, PNG (Figure 1), which is located in a disadvantaged urban area and administered by a non-governmental organization (HOPE Worldwide, PNG) on behalf of the National Capital District Department of Health. In addition to providing services to women and men from the general community, the clinic has an active community outreach and peer support program developed in collaboration with Family Health International (FHI) PNG that is designed to facilitate service uptake by people at increased risk of HIV/STI acquisition, particularly male and female sex workers and men who have sex with men. Clinic staff provided an overview of the study to potential participants as part of routine health talks (*tok save*) given at the start of each day to clients attending the clinic. Peer educators provided similar information to potential study participants through their community outreach activities. Those who expressed an interest in hearing more about the study and potentially taking part were invited to participate in preliminary group discussions at the clinic in which they were provided with more detailed information about the study by trained researchers and clinic staff. Clients who expressed an interest in study participation were then invited to take part in an in-depth interview (IDI), which took place at a location of their choice following the completion of informed consent procedures. This study location was selected because of the high proportion of women and men at increased HIV/STI acquisition risk who attend this clinic, including FSWs and their clients, and vulnerable individuals living in illegal urban settlement areas. Separate IDI guides were developed for interviews with women and men by the research team, in consultation with clinic staff. IDI guides included four thematic areas of inquiry: background information; sexual behaviour and sexual health; intravaginal and menstrual practices; and female-controlled methods of HIV protection. All interviews were conducted by trained, experienced and gender-appropriate interviewers in *tok pisin* (VF, HA).

Socio-demographic and sexual behavioural data, including experience of male and female condom use, were collected from all participants. To facilitate discussion on intravaginal hygiene and menstrual practices (IVP), women were invited to use copies of a hand-drawn template to indicate how they wash/clean their vulva and/or vagina, and to provide commonly-used, locally-appropriate names for anatomical structures by labelling the drawing. Templates were developed by the research team in consultation with clinic staff and piloted with volunteers from the clinic for appropriateness and acceptability prior to use. Women were also asked to comment on practices they had heard other women in the community were conducting and to indicate these using the template. Men were asked whether they were aware of their sexual partners conducting such practices or if they had heard about other women in the community conducting such practices, and to use the template to indicate these. Participants indicated IVP using marker pens, highlighter pens, biro and/or pencils provided by the study team and if literate, were asked to write the names of anatomical structures onto the sheet. Where participants were unable to read or write, interviewers wrote down participant’s comments / anatomical names on the sheet on their behalf, using English and/or *tok pisin* depending on the language used by individual participants.

Men and women were shown male and female condoms, which interviewers’ opened and encouraged participants to handle and comment on. Participants were asked to reflect on their experiences of condom use; the factors that had determined use in different contexts and behavioural scenarios; their personal views of condom acceptability; and their views about acceptability and use among women and men in the wider community.

**Figure 2:**
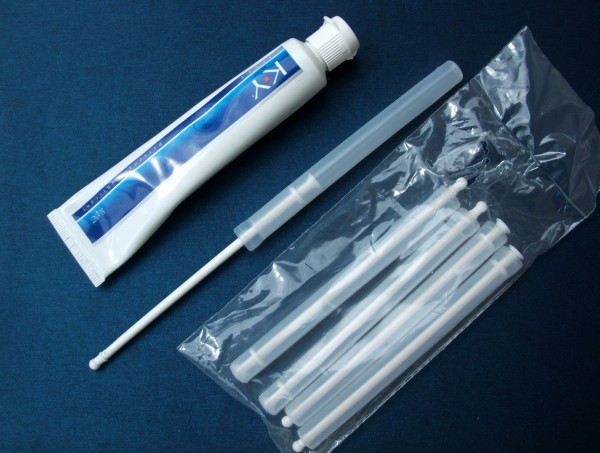
Products used to make ‘dummy’ microbicide gel and applicator.

**Figure 3:**
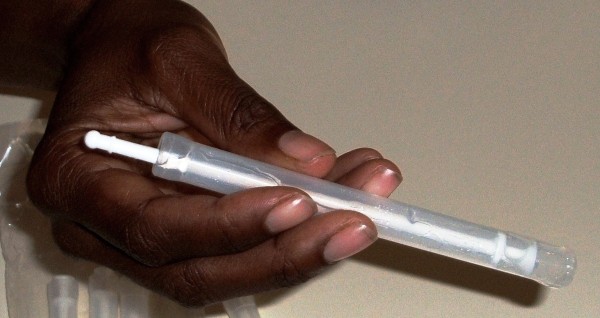
Demonstrating microbicide surrogate to study participants.

Interviewers introduced the concept of a vaginal microbicide to participants and demonstrated a surrogate microbicide gel and applicator, prepared prior to each interview by research staff using commercially available vaginal applicators that were pre-filled with 2-3mL KY Jelly® (Figure 2 and 3). This volume was selected based on earlier microbicide efficacy trials, in particular the MDP301 trial [4]. Interviewers demonstrated how such a product would be used in practice, based on information provided to MDP301 trial participants [73]. Participants were invited to handle the surrogate microbicide and to ask any questions they may have regarding future use of such a product.

***Interviewer:****‘Now I’d like to talk to you about new methods of protection being developed. We hope that women will be able to use these to protect themselves against HIV and sexually transmitted infections in future. These products are known as ‘vaginal microbicides’ and will probably be available in the form of vaginal gels or creams. We hope that an effective microbicide will become available in the next 3–5 years. I have here an example of what a vaginal microbicide gel might look like (***Hold up pre-loaded dummy applicator***)*. *In future, women could insert a gel like this into the vagina, either daily or just before each sex act, to protect them against HIV and sexually transmitted infections.” (***Demonstrate how applicator would be inserted into the vagina; different positions she could use for insertion; gel volume and consistency. Give her the applicator to handle. Encourage her to ask questions about microbicides***).*

Interviewers then asked participants to reflect on their views of personally using such a product in future, in the context of their individual sexual lives and reported IVP e.g. would they tell their husband / sexual partner(s) that they were using such a product or might they in some situations consider covert use? Similarly, they were asked to reflect on how such a product would be perceived and used by the wider community.

All interviews were digitally recorded, transcribed verbatim and where necessary translated from *tok pisin* (a lingua franca of PNG) into English at the PNG Institute of Medical Research (PNG IMR) in Goroka. All personal identifiers were removed from the interview transcripts and pseudonyms given to each participant. Following data cleaning and initial review of interview transcripts, a preliminary coding framework was developed by the research team to capture data relevant to each key thematic area of inquiry (e.g. respondents views on the frequency of IVP among women in their community were coded ‘IVP-freq-comm’; individual motivators for IVP were coded ‘IVP-mot-self’). The research team then hand-coded printed interview transcripts. The coding framework was periodically reviewed as data coding proceeded and additional codes and/or sub-codes added in an iterative data management process. Themes were derived from the data during preliminary analysis of the coded transcripts, which was led by senior members of the research team (LF, AV), prior to critical review and discussion with the wider group e.g. to seek alternative themes and explanations arising from the data. A summary of key findings and their interpretation was then drafted (AV, LF) and illustrative quotes selected from individual interview transcripts in order to exemplify specific issues and concepts that had emerged across the dataset.

Ethical approval was obtained from the Medical Research Advisory Committee (MRAC) in PNG and from the Human Research Ethics Committees of the University of Queensland and the University of New South Wales in Australia. All participants provided written informed consent in either English or *tok pisin*. All participants were advised that they could withdraw from the study at any time without prejudice. The study was conducted in accordance with the RATS guidelines on qualitative research (http://www.biomedcentral.com/ifora/rats).

## Results

Twenty-eight IDIs were conducted with women (n=16) and men (n=12) from diverse socio-cultural backgrounds (Table 1). The majority of participants (19/28) lived in urban settlements at the time of the study. Two women who self-identified as FSWs took part in this research.

**Table 1:** Socio-demographic characteristics of study participants

	**Women (n=16)**	**Men (n=12)**
**Age**		
Age range (mean)	16–42 y (25 y)	17-54 y (30 y)
**Marital status**		
Single	4	2
Married	10	9
Divorced	2	1
**Current residence**		
Port Moresby (settlement area)	12	7
Port Moresby (non-settlement)	3	5
Reside outside Port Moresby	-	-
**Province of origin / family origin**		
Central	2	1
Gulf	1	1
Milne Bay	-	1
Oro	-	1
Eastern Highlands	2	2
East New Britain	1	-
East Sepik	1	-
Simbu	5	2
Southern Highlands	1	4
Western Highlands	3	-
**Occupation**		
Unemployed	14	8
Skilled employed	-	2 (1 clerk; 1 teacher)
Unskilled employed	-	2 (security guards)
Commercial sex worker	2	-

### Traditional customs and norms relating to women’ bodies

Traditional customs and norms surrounding women’s bodies were described by both male and female participants. Such traditions were reported as common among people from communities in the Highlands of PNG, where menstruating women isolate themselves from male family members, not touching or preparing food, or having sexual intercourse, for fear that maternal blood could ‘contaminate’ or weaken men. Male participants drew on traditional stories of men being weakened in warfare and becoming sick if they ate food prepared by, or had sex with, menstruating women. Several men described providing support to their wives during their monthly menses, in order to support customary practices, for example:

“I’m an old man and at this time now. In the past when women have their menstruation like my wife, when having her flow and she cooks, I will not eat the food she cooked …the food will not taste good and we will vomit. She will not walk in front of me or close to where food will be prepared. She will stay on her own for until one or two weeks later she can hold or serve food. She can bring food from the garden but I will cook for the family. That is our traditional behaviour”. John, male

*“So when this thing* [period] *comes I use to tell my wife, let me cook, and you rest for four or five days”. Mego, male*

Women talked about the importance of *flow* of menstrual blood from the womb, and how flow could be ‘blocked’ by sexual intercourse during menstruation. Female participants described learning cultural practices from female family and village members. Some participants lamented the discontinuation of traditional customs, especially those who had moved away from their village. A number of participants described how they themselves did not follow all traditional cultural practices, for example, by engaging in sexual intercourse during menstruation.

*“The women when we have our monthly flow, we must not cook food and give to our family members especially our husbands. That is a very important thing. I will not cook food and give. I must not come close to the kitchen or the chair where my husband sits. I must stay outside and they will give me food. It’s a very big taboo for us to have sex with our husbands during that time …it’s a belief that when he goes to the village he will not have money, food or all these things* [if sex during menstruation occurs]*”. Pate, female*

### Intra-vaginal hygiene and menstrual practices (IVP)

**Figure 4:**
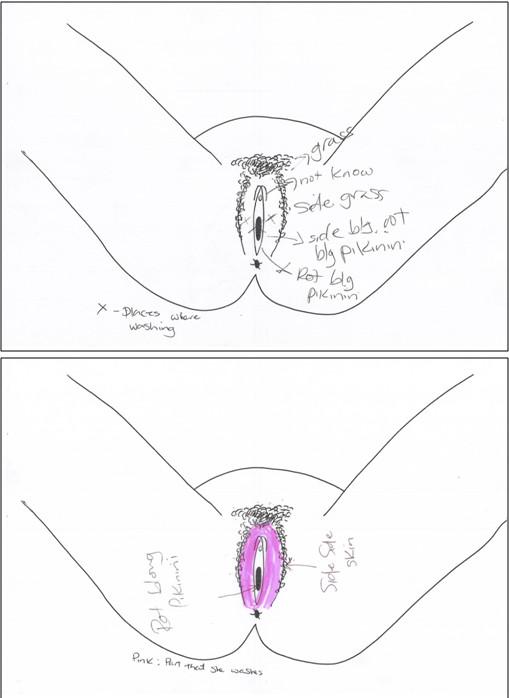
Example IVP templates completed by female study participants.

A diverse range of IVP were reported by female participants using the pre-tested template (Figure 4). Many anatomical structures were referred to using descriptive terms and did not appear to have specific names in *tok pisin* (e.g. *bean* for clitoris; *grass* for pubic hair) and there was no common term for vulva. Vagina was commonly referred to as *rot blong pikinini* [Lit: ‘baby’s road’] by both men and women; but *kan* used only infrequently e.g. among sex workers.

A typology or classification framework of IVP has recently been proposed, based on research conducted as part of a WHO Gender, Sexuality and Vaginal Practices (GSVP) Study, within which the results of the current study have been summarised (Table 2) [30].

**Table 2:** **Summary of intravaginal practices reported by women and men taking part in this study, using the classification proposed in the WHO Gender, Sexuality and Vaginal Practices Study **[29]^1^

**WHO Classification of Intravaginal Practices**	**IVP reported in this study**	**Motivators for IVP reported in this study**
External washing (cleaning vulva / external genitalia)	· soap and water	– to stop smell / remove dirt
		– to prevent sickness in the womb
		– to have children
		– to please sexual partners
Intravaginal cleansing (internal cleansing or washing of the vagina)	· ‘Klina’ laundry soap	– for genital cleansing
	· bath soap and water	– to help remove excess fluids
		– to please male sexual partners
		– to maintain reproductive health
External application (rubbing or placing products onto the vulva / external genitalia)	*Not reported*	*Not reported*
Intravaginal insertion (pushing or placing something inside the vagina)	· cotton, paper, tampons	– to absorb menstrual blood
	‘virgin soap’	– to tighten vaginal wall in order to increase male partner pleasure
	· ‘Klina’ laundry soap	– to induce abortion
		– to help release menstrual blood
		– for cleansing the vagina
	· crushed garlic	– to help ensure a healthy womb
		– to contract cervix after childbirth
Oral ingestion	· bark / leaves of specific trees	– to prevent pregnancy
	· ‘Klina’ laundry soap	– to induce abortion
Vaginal steaming or smoking (sitting above a source of heat on which water and herbs or oils are placed to create steam or smoke)	· Steaming (using boiled water infused with herbs / garlic /bark / leaves)	– to facilitate flow of menstrual blood and prevent blockages
	· Smoking (using burnt coconut leaves)	– to cleanse and tighten the vagina
Anatomical modification (“cutting and pulling” for modifying the vulva; restoration of the hymen)	Vulva piercing	– to increase sexual pleasure (male / female)

^1^IVP and motivators reported by a representative sample of those interviewed, unless otherwise indicated.

The majority of women in PNG described washing the vulva only with soap and water as part of their daily routine; in preparation for sex; and following sexual intercourse. Several women described cleaning inside the vagina using fingers and soap at these same times. Others reported cleaning inside the vagina using a hose connected to a tap; use of vaginal inserts (such as crushed garlic for improved genital health or ‘virgin soap’ for intravaginal tightening); customary ‘steaming’ practices associated with menstruation; and the use of material fragments, cloth, newspaper, baby nappies and sanitary towels to absorb menstrual blood. Unprotected sex during menstruation appeared common and was reported by both women and men.

Washing was undertaken for the purposes of hygiene, to avoid ‘bad smell’, to remove ‘dirt’, to prevent ‘sickness’, and to ‘protect the womb’. Female participants learnt about vaginal hygiene from their mothers and grandmothers as part of traditional rites of passage. For those living in urban localities, friends were the main source of information. It was acknowledged that urban residents had more access to water and hence washed more regularly than rural residents. Most described cleaning as a private matter not discussed with male partners, although a few participants did discuss IVP with their partners, with some male partners actively helping partners to wash.

*“No I soap my outside body and wash it… I don’t push it right in* [the vagina] *but just enough to wash it”. Seguto, female*

*“I don’t think all women do this because when I walk past them, I smell their body odour…they don’t wash it* [vagina] *properly…dirt is still there and it comes down on the underwear and bad air comes from it that causes the bad smell… Personally I fear of getting sick inside the womb so the way I wash myself is that, I open my two legs and I put my hand right inside. I put my hand right inside and wash it…not all the fingers but only one or two fingers…I put inside and soap it. I soap it, wash and rinse it properly. I open my legs like this and I wash it. I’m scared of diseases. So I wipe it with a towel when washing and clean it properly”. Napoga, female*

“..when they pass you it will smell and you will say ‘she must not have washed…she is smelly’…they must wash every morning and afternoon. It is good they keep their bodies and vaginas clean to protect themselves and to have children easily”. Mego, male

A number of intravaginal practices were associated with menstruation. The choice of menstrual products was associated with price, availability and cultural norms. Sanitary products were described as expensive and difficult to access. It was uncommon for women to use internal menstrual products. Participants who practiced traditional customs associated with menstruation discussed the importance of releasing menstrual blood and did not use tampons or other internal products for fear that these would ‘stop the flow’ of blood and cause sickness. To aid the flow of blood, a few women described traditional ‘smoking’ practices using burnt coconut leaves, and ‘steaming’, which involved sitting over hot water infused with herbs and garlic, or leaves from lemon, guava and avocado trees.

*“I think that when wearing modes* [sanitary pads] *or toilet roll and all this, it blocks the bad air back inside and by this I think it will cause sickness inside”. Napoga, female*

*“Some women tear off pieces of cloth and put it into their underwear or such. They can’t afford to buy* [sanitary products] *so, they use newspapers. Okay some women wear laplaps* [cloth wrap-around skirt]*. They just roll down or put it to their back. Women in the village wear these laplaps to stop themselves staining their trousers or skirts. Some women stay inside the house”. Kakana, female*

*“I pour the water* [herbal infusion] *into a bucket, sit down on the bucket and the steam will go inside. It will clean the dirt inside and you will see it in the water. After steaming, you will feel light and nice. When we wear pads, it blocks the bad blood inside. When you steam the blood will come outside …the dish will be full of it’ Napoga, female*

Several female respondents reported hearing of women cutting up soap bars (e.g. ‘Klina’ soap available in PNG) into small blocks and inserting them into the vagina prior to menstruation. When menstrual blood appears, the soap is washed out.

Many female participants reported sex during menstruation, often under pressure from their male partners, and despite cultural taboos relating to these practices and to the importance of menstrual exclusion among women from Highlands communities. Women and men from cultural groups that traditionally considered sex during menstruation taboo reported engaging in such practices, whilst recognising the conflict between their contemporary and customary beliefs. Several male and female respondents said they would not consider using a condom when having sex during menstruation due to concerns about the condom *‘blocking rubbish to come out’*:

“And when I have my period, he asks to use a condom but I use to say no. When he has sex with me during my menstruation time, it stops, I don’t have my period. He stops it and I get afraid of that because he puts back inside; the rubbish. So I will get sick, it worries me. Napoga, female

IVP for contraception and to induce abortion were also described. Eating the bark and leaves of a certain tree was discussed as a form of contraception, or a means to induce abortion e.g. in combination with the intravaginal insertion of soap. Others described tying ‘a *rope around the belly, and putting soap inside the vagina’* to cause abortion.

“Yeah they usually mix hot water with soap and egg and drink it… They drink it and it comes out like blood”. Dorothy, female

‘Virgin soap’ was discussed by female participants as an increasingly popular vaginal insert used by ‘mothers’ or ‘married’ women to tighten the vagina. The soap was seen as a proactive method to keep male partners faithful by making sexual intercourse more pleasurable for them. One participant who had tried the soap described a painful, burning experience:

*“They said when we women give birth to children, our muscles become weak but his soap will cause it to tighten the women’s muscles. …I bought* [virgin soap] *from one woman; they sold it for 5 Kina* [approx. USD 2.00]*. It was a small bit they sold and they said it was virginity soap. They said if wash with this soap, it will contract your muscles and you will become small and 16 again and when you have sex with your husband, he will really like it. So they convinced me and I bought one and used it only one time. I soaped myself and they told me to put it into the vagina for around 30 or 20 seconds and it just burned me. It really burned me and I just stopped and rinsed myself and I said that I won’t use it again”. Kakana, female*

In contrast to other settings [15,24,25], none of the women interviewed reported inserting lemon, lime, herbs or other substances (e.g. to increase or decrease vaginal lubrication) and both women and men were unaware of such use in the wider community. The use of vulval inserts was described by one female participant (*“they used to shave the hair of the vagina and put earrings along the sides”*), which she considered a practice carried out mainly by women who engage in sex work. Anecdotal accounts of such practices were also reported by health care workers at Nine-Mile Clinic.

### Female condom use and acceptability

The majority of men and women said they had not previously seen or handled a female condom but said they would consider using one in future, primarily with non-regular partners because they trusted their spouse or long term partner. The dominant sexual practice discussed was ‘straight sex’or ‘skin to skin’, that is, vaginal sex without a condom. Although some participants had used male condoms, mainly for contraception, few did so regularly and few used male condoms for protection against STIS/HIV. Condoms were described as being for unmarried people, or those not in ‘trusting’ relationships.

“We think that condoms are for promiscuous people… so we don’t think of using condoms: we do it skin to skin”. Kakana, female

*“We don’t usually use them* [condoms]*. He used to talk about using it* [a condom] *but I usually say ‘I am not your prostitute’ and ‘I have what kind of sick?’ and I used to get cross with him; chase him away from the house”. Napoga, female*

When shown a female condom in the interview context many participants were very interested in learning about them, especially female participants, some of whom were worried about their partner’s infidelity. Only three women reported using a female condom in the past, all of whom said that they would continue to use and to negotiate use with their partners in future.

### Vaginal microbicide gel acceptability

The majority of both women and men said that they would use a product like the surrogate gel and applicator to provide protection against genital infections, STIs and HIV, should a safe and effective product become available in future. Many participants said that they would use a future product because they thought it would not only provide protection against STIs and HIV, but also enhance the ‘health of the womb’ more broadly or ‘prevent us getting sicknesses like other kinds of infection or sexual disease or pregnancy’.

The majority of women and men interviewed in the study thought that future microbicide use would be most appropriate in perceived as ‘high-risk’ situations, such as sex with non-regular, transactional or commercial partners (‘*two Kina meri’*). Many respondents thought it unnecessary to use a microbicide with ‘trusted’ partners, as the following quotes illustrate:

“The sex workers who go around having sex, these kinds of women usually get the sick and give it to the men, so it’s good that they bring this medicine now so that it will help us, the married couple. These women have sex with our husbands and they spoil our married life. The married man will bring the sick, so it’s more good that the medicine will come and the women will use this, and the sick will not come into the married couple. I want this medicine to come”. Napoga, female

*“I won’t tell her* [my wife] *to use it because she trusts me and I trust her” Moko, male*

*“I trust him* [steady boyfriend]*…I will use it only with some other partners” Daisy, female*

Most women felt confident that they would be able to negotiate future vaginal microbicide use with their sexual partners but if necessary, would be prepared to use products covertly. Covert use was considered empowering for married women who were worried about their husband’s infidelity. Some female participants however, questioned covert use for fear of inciting male partner anger or violence.

“I like what you said about using this gel. You know I am a housewife and the man is working and he goes out. Sometimes when I insist to use a condom during sex he will say, ‘I’m not your boyfriend’ and he will throw it away. I like what you talked about the gel and my husband will not know about it if I use, knowing that he’s having affairs outside”. Pate, female

*“It’s my secret so I will insert it* [vaginal microbicide] *and we will have sex. I will not tell them because some have diseases and are thinking to give it to me. If I put this medicine in* [with their knowledge] *they will be cross, get upset and hit me.’ Dorothy, female*

When shown the surrogate microbicide most women agreed that the product would be easy to use, and could be applied in various geographical locations before sex. Some women expressed concerns that applicators might be difficult to use without initial counselling and support from health care staff; that incorrect use could result in vaginal trauma; that gel might leak out when urinating or standing up; that gel use could affect a woman’s ability to conceive.

“If we don’t put it in properly it might go right inside…might damage the vagina”. Dorothy, female

Many women asked questions regarding the ‘wetness’ that the gel would cause, and what the reaction of male partners might be to this, especially if gel were being used covertly. ‘Wetness’ was associated with menstruation or having an STI, and some female participants feared the reaction of male partners if extra wetness were perceived. One female participant however described how increased vaginal lubrication might make sex more pleasurable for women.

“Yeah when I insert this medicine inside and when we want to have sex, he will ask me about the wetness. He will ask me if I’m having my monthly menstruation”. Dorothy, female

“Some men when they want to have sex they prefer the woman’s vagina is dry and if this thing is put inside and it becomes watery, the men will ask the women to clean it and there’s no choice”. Seguto, female

“If our husbands don’t agree and we use this medicine, we put it and they thinks it is too greasy and they find out, this too will bring some problems into the family”. Kakana, female

One female participant questioned the use of the microbicide for fear that it would ‘block’ the flow of maternal blood, and cause sickness to the womb:

“…when we put this thing inside, it will block this monthly flow to come outside and will cause some kind of problem to the womb. It will go and damage the tube inside where the flow comes”. Daisy, female

Male participants were generally positive about the potential benefits of a vaginal microbicide for the health of their female partners and for their own protection against STIs/HIV, however two male participants suggested that the use of a vaginal microbicide might cause more ‘promiscuous’ sexual behaviours.

*“I think that we must not put it in public; we must give it to the people who come to the clinic only. If they want to use it they must use it themselves …putting it public will make them think that the medicine have come already so they can do it* [have sex] *and when they continue to do that it might increase the HIV rate in PNG. I saw in the newspapers that the HIV rate of Australia is 0.02% or something and PNG is 2% or 3% and up. We have a small population and they introduced condoms in public and this has made it worse so how will this* [microbicides] *reduce this?” Mego, male*

## Discussion

Intra-vaginal hygiene and menstrual practices (IVP) were diverse in nature, socio-cultural dimensions and motivators in this setting. A dynamic combination of traditional and contemporary practices were reported, with some women engaging in new practices that they recognised as being in conflict with traditional norms and beliefs(e.g. the use of ‘virgin soap’; sex during menstruation). Broad reproductive health concerns (maintaining genital cleanliness, removal of ‘dirt’, preventing ‘bad smell’, maintaining a ‘healthy womb’) appeared to be more important motivators of vaginal practices than specific concerns focussed on enhancing sexual pleasure or on the prevention of HIV and STIs. The WHO Gender, Sexuality and Vaginal Practices (GSVP) Study included a household survey on IVP conducted among 3610 women aged 18–60 years in Mozambique, South Africa, Indonesia and Thailand [29,30], and found that the prevalence, motivators and contexts of vaginal practices varied markedly between countries. IVP in participating African sites appeared to be motivated primarily by a desire to maintain male partner commitment and to increase male and female sexual pleasure. Conversely, in participating sites in Asia, genital hygiene and femininity were primary motivators. Researchers in a variety of settings [15,24,29,30,33,74] have similarly distinguished ‘sexual-related’ and ‘hygiene-related’ IVP, and highlighted the potential of vaginal washing, insert and other practices (conducted at different time points, and driven by different motivators), to modulate susceptibility to HIV/STI acquisition and transmission. For example, women who use lime or lemon juice intravaginally (e.g. as a traditional contraceptive; for protection against genital infections; or for enhanced male sexual pleasure [24,34,75]) are likely to be at increased risk of cervical dysplasia, cervical epithelia disruption and HIV acquisition, based on recent laboratory and clinical trials data [76-80]. The use of soaps, detergents, and other products used as part of daily genital hygiene or carried out before and/or after sex, could potentially result in vaginal irritation, inflammation or epithelial disruption, thereby increasing infection risk. A recent systematic review and meta-analysis of 15 studies in Africa and the USA concluded that intravaginal practices do not appear to protect women from STIs, genital infections or HIV, and that there is inconclusive evidence whether some practices may increase acquisition risk [22].

Depending on their nature and timing, vaginal practices also have the potential to negate the protection afforded by vaginal microbicides, either by mechanical means (e.g. microbicide gel is diluted or washed out) or through chemical interaction (e.g. vaginal insert chemically reacts with microbicide product or alters intravaginal environment sufficiently to reduce product effectiveness). Although we attempted to clarify the temporal relationship between IVP and vaginal sex in this study, it was not possible to establish precise estimates during in-depth interviews with participants. Vaginal insert practices appeared uncommon in this setting and no respondents reported the insertion of lemon or lime juice. Sex during menses was however common and has the potential to increase the risk of HIV infection due to blood exposure (particularly transmission to uninfected male partners [81-85]), and to reduce microbicide effectiveness due to altered vaginal pH and other physiological changes [86-88]. Further research is warranted, using combinations of behavioural research methods as utilised in other settings [24,74,89-91], to better understand these inter-relationships and their implications for future microbicide uptake, adherence and effectiveness in PNG.

Notional acceptability of vaginal microbicide gels for HIV/STI prevention was high among both women and men in this study. Microbicide acceptability was perceived within a broad reproductive health framework i.e. that use would promote a ‘healthy womb’ or help ‘prevent sickness or pregnancy’, in addition to preventing HIV. This perception was held by women and men and is in contrast to advice provided by the research team. Conversely, one female respondent was concerned that gel could ‘block’ menstrual flow and cause sickness in the womb. Many women and men thought that microbicides would best be used by sex workers and considered use between ‘trusted partners’ unnecessary. Most women were confident that they would be able to negotiate gel use with their sexual partners in future and many were prepared to use microbicides covertly should their partner refuse. Other women were concerned that increased vaginal ‘wetness’ would make truly covert use difficult and could expose them to increased risk of physical or sexual violence. There were mixed views on the potential of microbicide gels to modify sexual pleasure for women and their male partners. Some women reported that their partners preferred ‘their vagina dry’ and that men would ‘ask women to clean it [out]’ should they use gel; one woman reported that increased vaginal lubrication would make sex more pleasurable for women. Women were confident that they could use a product similar to the surrogate microbicide in future but some were concerned that initial use might be problematic without appropriate counseling and support from health workers. These findings clearly have implications for future microbicides research and for the introduction of safe and effective vaginal microbicides for HIV prevention in PNG.

Low levels of awareness and lack of experience using female condoms are cause for concern, but also represent an important opportunity for comprehensive HIV prevention programs in PNG, given that notional acceptability was high among women and men in this study. Further research to clarify in-use acceptability and adherence of female condoms, including gendered sexual dynamics, socio-cultural contexts and motivators influencing use, are warranted to inform female condom promotion programs in this setting.

A challenge in countries such as PNG that have great geographic, linguistic and cultural diversity is to develop culturally-nuanced and innovative strategies for HIV prevention. This study has shown that women and men from a variety of socio-cultural backgrounds, who are at increased risk of HIV/STI acquisition, are notionally supportive of vaginal microbicides for HIV prevention in this setting. The need to engage male partners in microbicides research and in strategies for future product introduction has been recognized [13,27] and is essential in countries such as PNG where sexual dynamics are particularly male dominated. It remains unclear how other microbicide formulations currently in development (such as intra-vaginal rings, dispersible vaginal tablets and films [8-10]) would be perceived and most effectively incorporated into people’s individual sexual lives in this setting. In-use acceptability research planned by our group will address these issues in future.

What are the implications of our research findings for HIV prevention in PNG and other Asia-Pacific countries? Firstly, our findings need to be interpreted with caution given the modest sample size, purposively selected from a population of women and men attending an urban sexual health clinic in a disadvantaged area of Port Moresby, making generalisability problematic. This population is however considered representative of people at increased risk of HIV and STI acquisition in PNG, a key potential target group for the future introduction of vaginal microbicides and other biomedical prevention technologies. Other limitations are that the notional acceptability of alternative vaginal microbicide formulations and dosing regimens were not evaluated in this study (including coitally-independent products such as intravaginal rings); and because this research was conducted prior to the successful CAPRISA 004 trial result becoming available, gel acceptability was not evaluated in the knowledge that a safe and effective vaginally-administered product had been identified. Although biomedical prevention technologies such as male circumcision and vaginal microbicides are expected to play an important role in comprehensive HIV prevention strategies in high-burden settings, their role in moderate prevalence settings is less clear. A key challenge having investigated notional acceptability and the determinants of acceptability will be to decide how future interventions could best be implemented in order to ensure the greatest public health impact. Population-level implementation of a moderately-effective vaginal microbicide is likely to be less cost-effective in lower burden settings than targeted introduction among those most at-risk of HIV acquisition [92-94], such as women in HIV sero-discordant relationships or female sex workers [95]. The ethical and moral hazards of not making such products widely available to all women clearly also need to be considered, particularly in countries where women’s sexual agency may be very limited, but should be balanced against potential risks such as condom migration [12,14] and the emergence of antiretroviral drug resistance [96]. Comprehensive guidance on these issues for low and moderate burden settings is not currently available but is urgently required to guide HIV prevention policy outside Africa. Complementary health systems research [97,98] and epidemiological mathematical modeling [92,93] being conducted by our group are assisting policy makers and development partners in PNG to develop evidence-based policy. This approach may represent a model for other moderate-burden countries considering a combination of biomedical prevention technologies for HIV prevention.

## Conclusions

Notional acceptability of a vaginal microbicide gel for HIV prevention was high among women and men at increased risk of HIV and STI acquisition attending a sexual health clinic in PNG. Further research is warranted to investigate the in-use acceptability and adherence of different microbicide formulations in this setting, and to establish the role of vaginal microbicides and other biomedical prevention technologies in countries experiencing moderate-burden HIV epidemics.

## Consent

We obtained verbal consent from all those in the photograph.

## Competing interests

The authors declare that they have no Conflicts of Interests.

## Authors’ contributions

AV, LF and AK conceived, designed and coordinated the study, conducted the literature review and drafted the manuscript. JN, JM, JS, MK, PS and JK participated in the design and coordination of the study and helped draft the manuscript. VF, HA, JS participated in the design and coordination of the study, conducted in-depth interviews and focus group discussions, and helped draft the manuscript. All authors have read and approved the final manuscript.
